# Maize *ARGOS1* (*ZAR1*) transgenic alleles increase hybrid maize yield

**DOI:** 10.1093/jxb/ert370

**Published:** 2013-11-11

**Authors:** Mei Guo, Mary A. Rupe, Jun Wei, Chris Winkler, Marymar Goncalves-Butruille, Ben P. Weers, Sharon F. Cerwick, Jo Ann Dieter, Keith E. Duncan, Richard J. Howard, Zhenglin Hou, Carlos M. Löffler, Mark Cooper, Carl R. Simmons

**Affiliations:** ^1^DuPont Pioneer, 7300 NW 62nd Ave, Johnston, Iowa 50131, USA; ^2^DuPont Pioneer, 200 Powder Mill Road, Wilmington, Delaware 19803, USA

**Keywords:** Maize hybrid, native allele variation, transgene by environment interaction, transgene efficacy, heterosis, *ZAR1*, *Zea mays*.

## Abstract

Crop improvement for yield and drought tolerance is challenging due to the complex genetic nature of these traits and environmental dependencies. This study reports that transgenic over-expression of *Zea mays AR*GOS1 (*ZAR1*) enhanced maize organ growth, grain yield, and drought-stress tolerance. The *ZAR1* transgene exhibited environmental interactions, with yield increase under Temperate Dry and yield reduction under Temperate Humid or High Latitude environments. Native *ZAR1* allele variation associated with drought-stress tolerance. Two founder alleles identified in the mid-maturity germplasm of North America now predominate in Pioneer’s modern breeding programme, and have distinct proteins, promoters and expression patterns. These two major alleles show heterotic group partitioning, with one predominant in Pioneer’s female and the other in the male heterotic groups, respectively. These two alleles also associate with favourable crop performance when heterozygous. Allele-specific transgene testing showed that, of the two alleles discussed here, each allele differed in their impact on yield and environmental interactions. Moreover, when transgenically stacked together the allelic pair showed yield and environmental performance advantages over either single allele, resembling heterosis effects. This work demonstrates differences in transgenic efficacy of native alleles and the differences reflect their association with hybrid breeding performance.

## Abstract (precis)

A single transgene *ARGOS1* positively impacts yield of field-grown hybrid maize. Two predominant alleles from elite hybrid breeding germplasm differed in transgene efficacy, but both alleles combined in a transgenic stack outperformed each alone, consistent with a single-locus heterotic effect

## Introduction

Engineered crops with traits such as insect control (Estruch *et al.*, 1997; Kumar *et al.*, 2008) and herbicide tolerance (Dill *et al.*, 2008; Duke, 2011) are now widespread and have successfully helped to protect or stabilize crop yield. There is interest in the expanded use of biotechnology tools to complement breeding and impact complex agronomic traits such as grain yield, drought-stress tolerance, and nitrogen use efficiency. Such agronomic traits are considered quantitative in nature, controlled by multiple genes with usually small individual effects, and are also environmentally influenced. Transgenic testing, however, is still usually limited to testing one or a few genes at a time.

Conventional breeding has raised yield in maize year after year for decades (Duvick, 1999, 2001, 2005). Breeding concepts for testing and genetic information in the resulting improved germplasm may be useful guides for transgenic crop improvement. A major influence of traditional breeding is the selection and reshaping of allelic diversity in germplasm (Feng *et al.*, 2006; Hufford *et al.*, 2012; Jiao *et al.*, 2012). Elite germplasm and functional genetic variation for target traits are the key resources for breeders to continue developing higher yielding crop varieties. In a hybrid crop, such as maize in the U.S. Corn Belt, the germplasm has been organized into complementary parental heterotic groups. Parents of new hybrid combinations exhibiting improved traits and yield utilize the diversity that has been generated between the heterotic groups. To date, there has been little understanding of relationships between transgene effects and natural variation in elite germplasm and much less in relation to heterotic group partitioning of allelic combinations. Yet, such information could be a valuable resource for synergistic crop improvement by transgenesis or cisgenesis and conventional breeding.

The success in improving yield through breeding has mostly relied upon selection within the target population of environments (TPE), a set of environment ‘types’ within a geographical region wherein the varieties or hybrids will be released (Löffler *et al.*, 2005; Cooper *et al.*, 2006). Genotype-by-environment interaction for yield and complex agronomic traits is a well-known important factor in crop breeding (Beavis and Keim, 1996; Malosetti *et al.*, 2004; Vargas *et al.*, 2006; Boer *et al.*, 2007). In biotech crop improvement, consistent transgene efficacy across broad environmental conditions, as demonstrated in traits such as insect and weed control, is anticipated to be less likely to apply to complex agronomic traits known for their environmental influence. Therefore, for these traits, understanding the transgene-by-environment interactions will be crucial, just as it is important for genotype-by-environment interactions in traditional crop breeding.

Genes related to organ size control have been referred to as intrinsic yield genes (Busov *et al.*, 2008; Krizek, 2008; Gonzalez *et al.*, 2009). In this study, the *ZAR1* (*Zea mays ARGOS*) gene, the putative maize orthologue of the *Arabidopsis* ARGOS (*A*uxin *R*egulated *G*ene involved in *O*rgan *S*ize) gene (Hu *et al.*, 2003), was transgenically over-expressed in hybrid maize to evaluate its potential for improving yield. The aim was to test the effects of a single transgene on complex traits in maize, such as grain yield and drought tolerance, and on the difference in transgene efficacy of natural allele variants utilized by traditional breeding.

## Materials and methods

### Plant materials and tissue sampling

All inbred lines were from maize breeding germplasm of DuPont Pioneer (hereafter Pioneer). Hybrid SS1/NS1 (Pioneer hybrid 3394) is a commercial hybrid (Duvick *et al.*, 2004; Guo *et al.*, 2004, 2006). The parental inbred SS1 is an Iowa Synthetic Stiff Stalk (SS) line; the NS1 inbred is a Non Stiff Stalk (NS) line (Labate *et al.*, 1997; Feng *et al.*, 2006). For *ZAR1* native allele specific expression analysis, the same tissue samples and methodology were as described by Guo *et al.* (2004). Three biological replicate samples were used, and three whole seedlings were pooled as one RNA sample replicate. Immature ear (pre-pollination) tissue was sampled from field grown V19 stage plants (Guo *et al.*, 2006). Immature primary ears harvested from three individual plants were pooled as one replicate. Tissue for transgenic gene expression analysis was sampled by leaf punches from V6 stage plants. Leaf punches were collected from five plants randomly sampled from one row for each event. All harvested tissues were frozen immediately in liquid nitrogen and stored at –80 °C.

### T-DNA constructs, maize transformation, and transgenic plant production

The T-DNA constructs and plant transformation method have been described previously (Unger *et al.*, 2001; Cigan *et al.*, 2005). GATEWAY TECHNOLOGY (Invitrogen) was used for vector construction. The expression cassette contained the promoters, the full-length cDNA sequences, and the PINII terminator in pENTRTM/D-TOPO vector (Invitrogen). For transgenic *ZAR1* heterozygous allele testing, the genomic fragments contained approximately 1.5kb upstream of the start codon for the native *ZAR1* allele’s promoters. Due to haplotype differences in the promoter regions, however, 1724bp was ultimately obtained for NS1 and 1376bp for SS1 (see Supplementary Fig. S6 at *JXB* online). The plasmid also contained a red emitting fluorescent protein (Ds-Red, Clontech, Mountain View, CA), driven by an aleuron-specific promoter Prom_*HvLTP2*_ (Opsahl-Sorteberg *et al.*, 2004) to serve as a visible marker for transgene expression. In vectors where the CaMV 35S enhancer (Hayashi *et al.*, 1992) was incorporated, it was inserted upstream of the promoter in the forward orientation. The co-integrated JT vectors were introduced into *Agrobacterium* strain LBA4404 and used to transform embryos of either Hi-II maize (*Zea mays*), or an inbred line (PHWWZ) from Pioneer as described previously (Unger *et al.*, 2001; Cigan *et al.*, 2005; Guo *et al.*, 2010). Typically, 20 independent transgenic events were generated, and ten events with a single copy of the transgene and confirmed transgene expression were selected and advanced for crosses to wild type (non-transgenic) plants and further characterization.

Transgenic plants derived from the primary transformed T_0_ lines were backcrossed to the wild type inbred (non-transgenic) for three generations (for Hi-II maize transformation, the initial *ZAR1* SS1 transgenic tested in year 2007), or one generation (PHWWZ inbred transformation) to obtain transgenic (inbred) plants and non-transgenic null segregants (for all the other *ZAR1* transgenics) (Guo *et al.*, 2010). Transgenic (and non-transgenic null control) hybrids were produced for yield testing by hybridization of transgenic plants with a non-transgenic inbred. Transgenic and their non-transgenic sib (null control) were identified by the Ds-Red marker.

### Microscopic counts of leaf epidermal cells

The same microscopic analysis was used as described by Guo *et al.* (2010). Briefly, tissue was collected from plants grown in the greenhouse. A 2×2cm section was cut from the midpoint of leaf number eight.

### Field experiments

#### Phenotypic measurements 

Field experiments for phenotypic measurements were conducted in 2006 at Johnston, Iowa. The experimental design was nested with a set of transgene and null control, three replications, and 2-row plots (4 m row length) per event. All experiments were thinned (from 20, 30, and 40 plants per row) at the V3–V4 stage to 6, 15, and 24 plants per row, respectively, to achieve corresponding densities of 10 000, 25 000, and 40 000 plants per acre. Early stand counts were recorded and the leaves were marked on selected plants to determine the growth stage during the season. Biomass data were determined by harvesting four plants per 2-row plot per sampling date. Tissues were dried in a Blue M 146 standard mechanical convection oven to 0% moisture and then weighed. Leaf area was measured by removing the leaves from the plant and running them through a LI-3100 leaf area meter (Li-Cor Biosciences, Lincoln, NE, USA). Yield component data were determined by harvesting five representative ears per plot.

#### Yield testing 

Yield trials under high yielding environments were set up at four or five locations with four replications per location. Yield trials under drought stress conditions consisted of water deprivation treatments applied at flowering and grain filling (six to eight replications per treatment). Experimental designs were an incomplete block. The transgenic events were compared to null segregants bulked together from each of the individual transgenic events in the experiment. Statistical differences were determined at *P* <0.10.

#### Native variation and association 

Haplotypes were defined by considering SNPs and indels on a segment of approximately 400 bps within a locus (Beló *et al.*, 2008). Haplotypes for *ZAR1*, haplotypes 1 and 2, were examined within a subset of 424 inbreds to determine the allele frequency in elite breeding germplasm, and macro-haplotype analysis was conducted in a subset of 245 inbred lines that all carry haplotype 1 in the NS and haplotype 2 in the SS heterotic groups, respectively. For detailed experimental methods, see the Supplementary data, Association experiments, at *JXB* online.

### Statistical analyses

#### Phenotypic measurements 

Data were subjected to ANOVA using the general linear model (SAS Inst.), considering entries as fixed and replicates as random factors. Duncan’s *t*-test was used to establish the mean differences between transgene positive and negative nests. Mean differences were separated by the least significant test (LSD 0.05) when the *F*-tests were significant (*P* <0.05).

#### Association analysis 

Mixed linear model analysis of the BLUPs was performed to test the association of the *ZAR1* gene with trait phenotypes (Boer *et al.*, 2007; van Eeuwijk *et al.*, 2010). Marker genotypes for *ZAR1* were available on 218 (out of 234 NS) and 121 (out of 133 SS) individuals. The markers had multiple alleles and were treated as random effects in our model. To control for genetic background effects not attributed to *ZAR1*, co-factors were identified using a combination of association analysis and single marker analysis for 10 000 genome-wide marker loci available on the entries grown in our field experiment. This procedure identified a subset of markers that showed a significant (*P* <0.1) association with each trait. Additional information on analysis methods can be found in the Supplementary data, Association experiments, at *JXB* online.

#### Transgene-by-environment interaction analysis 

Yield and weather data from three years (2007, 2008, 2009) were used in the transgene-by-environment interaction analysis. Environments were classified based upon environmental factors in key vegetative and developmental stages including different mean temperatures, total solar radiation, and drought stress index generated from a crop model (Löffler *et al.*, 2005). Daily temperatures, rainfall, and solar radiation were observed and collected from weather stations during the growing seasons. Major environmental types that occur in the US Central Corn Belt include (i) Temperate Dry, which features as hot and dry; (ii) Temperate, which has low levels of abiotic stress; (iii) Temperate Humid, which has relatively lower temperatures and solar radiation compared with Temperate, and (iv) High Latitude, even cooler temperatures (Löffler *et al.*, 2005). Correlation analysis of the transgene efficacy of *ZAR1* under different environmental classes was done by using the method of Dean and Voss (1999). Key environmental factors that correlated with the transgene efficacy were identified by screening across plant developmental stages using a standard partial least squares methodology.

#### RNA isolation and RT-PCR 

RNA extraction, DNAse treatment, cDNA synthesis, and transcript quantification were conducted as described by Guo *et al.* (2010). For *ZAR1* allele specific expression analysis, RT-PCR primers (see Supplementary Table S1 at *JXB* online) were designed such that they would amplify both alleles simultaneously and flank an indel in the 5’ UTR region between SS1 and NS1, yielding a 525bp product for SS1 and a 464bp product for NS1, allowing physical separation on a 1.5% agarose gel. Primers were designed in conserved regions between the alleles for non-biased amplification. RT-PCR parameters (DNA quantity of template and primers, and the number of PCR cycles) were optimized by following a similar protocol as described by Guo *et al.* (2004, 2010). For Real-Time PCR analysis, the Taqman reverse transcription kit (Applied Biosystems) was used as described by Guo *et al.* (2010). Transcript levels of *ZAR1* were measured relative to the endogenous reference eIF4g, a maize eukaryotic initiation factor gene (GenBank NP_568534) with the DCt method as described by the manufacturer. The primers used can be found in Supplementary Table S1 at *JXB* online.

Plant germplasm and transgenic material will not be made available except at the discretion of the owner and then only in accordance with all applicable governmental regulations.

## Results

### The *ZAR* gene family

With interests in studying the class of genes that affect plant and organ size, the maize homologues of the *Arabidopsis* ARGOS gene were identified and named as *ZAR* for *Zea mays ARGOS*. *ZAR1–9* were initially identified by homology to sequences in the public and proprietary databases. Subsequent analysis of *ZAR1* allelic variation in the germplasm revealed that *ZAR2* is an allele variant of the *ZAR1* gene, thus the maize gene family contains eight gene members in the B73 genome draft. The genomic and chromosomal locations of the gene family members are summarized in Supplementary Table S2 at *JXB* online. *ZAR1* is the closest orthologue to the *Arabidopsis* ARGOS gene. The family members share overall low sequence homology except for a conserved core domain region (see Supplementary Fig. S1a at *JXB* online). All the *ZAR* genes encode relatively small proteins, ranging from 64 to 152 amino acids. They also have diverse tissue expression patterns based upon our Solexa RNA profiling database (see Supplementary Fig. S1b at *JXB* online). *ZAR1* is expressed in diverse tissues, but showed the highest expression here in root and pericarp tissues (alleles not distinguished here). While some gene family members are expressed at a high level and in multiple tissues (*ZAR3*), others are fairly specific to tissues: *ZAR4* and *ZAR7* are mainly expressed in the root, *ZAR8* is mainly expressed in the kernel tissues, and *ZAR9* expression is pollen preferred (see Supplementary Fig. S1b at *JXB* online).

### Transgenic over-expression of *ZAR1* enhances maize plant and organ growth

**Fig. 1.**
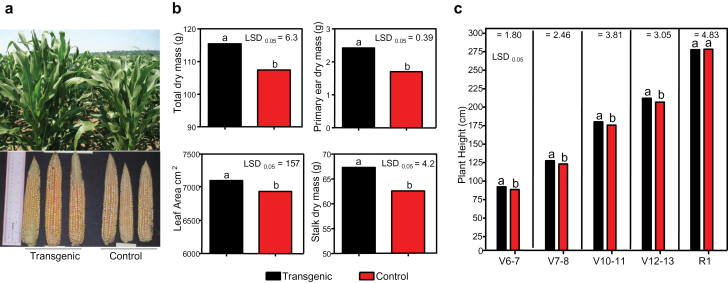
*ZAR1* transgene effects in maize plants. (a) Inbred transgenic plants over-expressing *ZAR1* (Pro_*ZmUBI*_:*ZAR1*) grown in the field: plants and immature ears during the growing season are shown. (b) Dry mass of whole plant, stalk, primary ear and leaf area at the V16 stage of hybrid plants over-expressing *ZAR1*. Black bars: transgenic; red bars: control: null segregant of the transgenic plants. (c) Plant height of hybrid at each developmental stage is an average of five plants per replicate, three replicates per event, and nine events in total. Plant density is at 32 000 plants acre^–1^. Different letters ‘a’ versus ‘b’ indicates a statistically significant difference at the given LSD.

*ZAR1* was constitutively over-expressed in maize by using the maize ubiquitin promoter (Pro*ZmUBI*). The transgenic plants showed enhanced vegetative and reproductive growth in the field in both inbred (Fig. 1a; see Supplementary Fig. S2 at *JXB* online), and hybrid plants (Fig. 1b), as measured by increased stalk, ear, and total dry biomasses, and leaf area. The enhanced plant growth was due to faster growth rate, not extended growth period, as the transgenic plants showed increased growth during development, and became fully grown faster than the non-transgenic controls (Fig. 1c). This differs from what was reported in *Arabidopsis*, where the larger plant and organ size resulting from ARGOS over-expression was attributed to an extended growth period, and thus delayed flowering (Hu *et al.*, 2003). Measurements of transgenic maize inbred plants grown in the greenhouse showed increased plant height, primarily resulting from increased upper internode length of 6th and above, but internode number was unchanged (see Supplementary Fig. S3 at *JXB* online). The increased internode length in transgenic inbred plants grown in the greenhouse was not observed in the field. This could be due to different growing conditions between greenhouse and the field, such as light, temperature, and day length. Microscopic analysis of maize leaf epidermal cells of inbred plants grown in the greenhouse indicated that the cell number (and stomata) counts per unit area remained unchanged or were slightly higher in the transgenics compared with the non-transgenic controls (see Supplementary Fig. S4 at *JXB* online). This indicates that *ZAR1* increased plant and organ size primarily through increasing cell number as cell size did not increase.

### Over-expression of *ZAR1* improves traits related to drought-stress tolerance

**Fig. 2.**
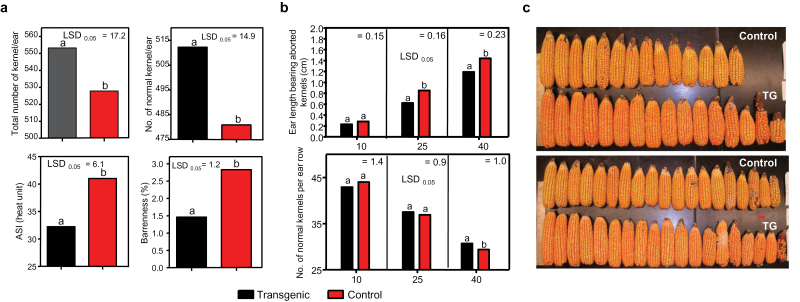
*ZAR1* transgene effects on traits relevant to drought-stress tolerance in maize hybrid plants. Transgenic hybrid plants over-expressing *ZAR1* (Pro_*ZmUBI*_:*ZAR1*). (a) Number of kernels per ear (upper left), total number of normal kernels per ear (the aborted or not fully developed kernels excluded) (upper right), ASI in GDU (lower left), and barrenness in the percentage of plants that did not produce any ear (lower right). Planting density was at 32 000 plants acre^–1^. (b) Tip kernel abortion under different planting densities: the length of the ear/cob carrying undeveloped kernels (upper panel); the number of fully developed kernels per ear row (lower panel): *x*-axis: population density (×1000 plants acre^–1^). Black bars: transgenic; red bars: control: null segregant of the transgenic plants. (c) Ear phenotype showing tip kernel abortion. Ears were harvested from all plants of the entire plot row. Different letters ‘a’ versus ‘b’ indicates a statistically significant difference at the given LSD.

Further characterization of transgene effects on other agronomic traits in hybrids showed that the *ZAR1* transgene improved several traits related to drought stress tolerance such as reduced tip kernel abortion, anthesis silking interval (ASI), and barrenness (Fig. 2). The effect on these traits became more pronounced when the plants were grown under higher plant density. As shown in Fig. 2b, the increase in total number of kernels became statistically significant as the plant population increased from 10 000 to 40 000 plants per acre, while not apparent at 10 000 plants per acre. The inbred transgenic plants showed enhanced primary and secondary ear growth (see Supplementary Fig. S2 at *JXB* online). These traits indicated enhanced plant growth and vigour, and tolerance to abiotic stresses such as drought and high plant density which prompted yield testing for drought-stress tolerance.

### The *ZAR1* transgene affects hybrid yield and exhibits transgene by environment interactions

**Fig. 3.**
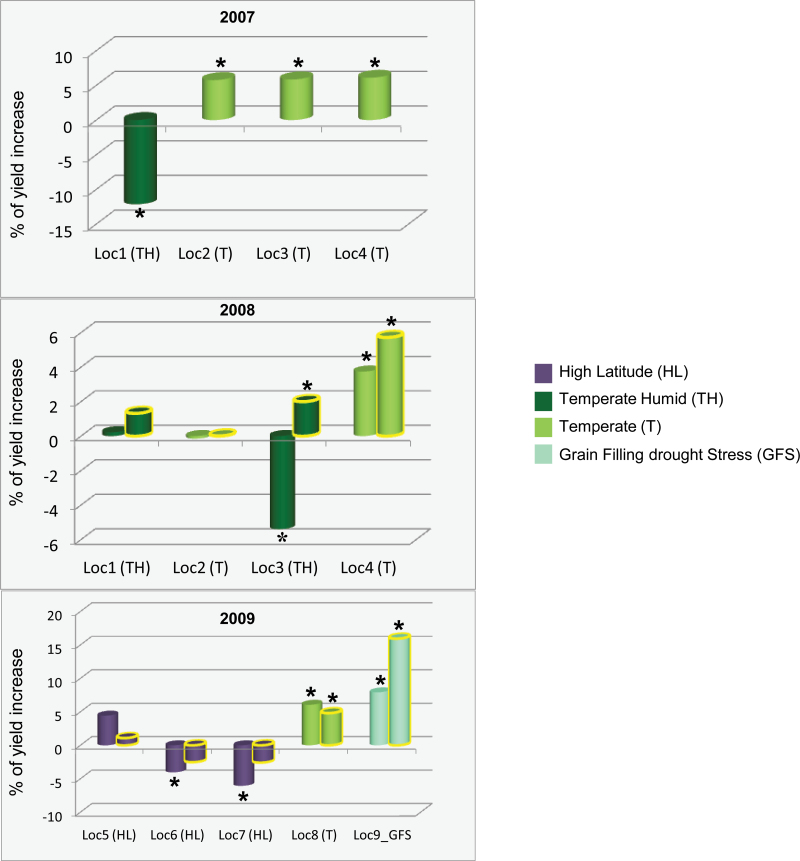
Yield difference of transgenic hybrid of *ZAR1* allele variants relative to control in multiple locations, environment classes, and years. Transgenic hybrids (Pro_*ZmUBI*_:*ZAR1*), SS1 (bar with no outline) or NS1 (bar with outline) protein coding allele variants, respectively) were yield tested in three years and four to five locations each year. Each location is colour-coded for the environment classes, * indicates statistically significant (*P* <0.1); *x*-axis, location and environmental classes, *y*-axis, per cent of yield increase of the transgenic (Pro_*ZmUBI*_:*ZAR1*) relative to the non-transgenic null segregant. Yield data were based upon six insertion events (2007), and 10–20 insertion events (2008 and 2009), respectively. Loc1: Linn, IA; Loc2: York, NE; Loc3: Tipton, IN; Loc4: Bureau, IL; loc5: York, NE; Loc6: Saline, MO; Loc7: Van Buren, IA; Loc8: Gibson, IN: Loc9: Yolo, CA. Locations are by U.S. County, State.

Transgenic maize hybrid plants over-expressing the *ZAR1* gene (protein coding region, SS1 allele) were yield-tested in 2007 at four field locations across the U.S. Central Corn Belt. The transgenic hybrid exhibited significant yield increase in three locations, but significant yield reduction in one location compared with the non-transgenic control. The second year (2008) of yield trials again showed a significant yield increase or decrease depending upon the location (Fig. 3; see Supplementary Table S3 at *JXB* online). *ZAR1* was initially cloned from a Stiff Stalk (SS, female heterotic group) inbred line (SS1 allele). A NS1 allele, which was present in most Pioneer Non Stiff Stalk (NS, male heterotic group) lines, was further isolated (protein coding region) and introduced to transgenic testing at the same locations as the SS1 allele in 2008. The transgenic *ZAR1* NS1 allele exhibited similar patterns of transgene effect on hybrid yield among these locations, but with less negative effects or yield reduction than the SS1 allele transgenic in those locations (Fig. 3; see Supplementary Table S3 at *JXB* online). These data indicated a functional difference between these two *ZAR1* alleles.

The significant yield increase or decrease of *ZAR1* (SS1 allele) transgenic hybrid plants was not constant in the same field locations between the two years. Since a given location may experience different environments across years, this result prompted the investigation of the transgene-by-environment interactions. Using the EnClass® system each test location was classified in 2007 and 2008 based upon environmental variables including rainfall, daily temperature, and cumulative solar radiation during the corresponding seasons (Löffler *et al.*, 2005). This process identified four major environment classes represented by the test locations across those years: Temperate Dry (TD), Temperate (T), Temperate Humid (TH), and High Latitude (HL), which are in the order of hotter, drier, and more solar radiation to cooler, more rainfall, and less solar radiation. Under these different environmental conditions, the *ZAR1* transgenics tended to exhibit significant yield increase under TD or T environments. The negative transgene effects were associated with the TH or HL environments (Fig. 3).

Because of the positive transgene effect on yield under hotter and drier environments, and also the previously observed effects on drought-stress-related traits (Fig. 2), a drought-stress treatment during the grain-filling stage (GFS) was included in 2009 yield testing (Fig. 3). Both the SS1 and NS1 alleles of the *ZAR1* transgene had a positive effect on yield under GFS and T environments and a negative effect under the HL environments. Consistent with the yield data in the second year, the NS1 allele performed better or had less negative effects on yield than the SS1 allele under the HL environments. These data confirmed the transgene efficacy, its relationship with environmental conditions, and the difference between the SS1 and NS1 alleles in both years (Fig. 3; see Supplementary Table S3 at *JXB* online).

**Fig. 4.**
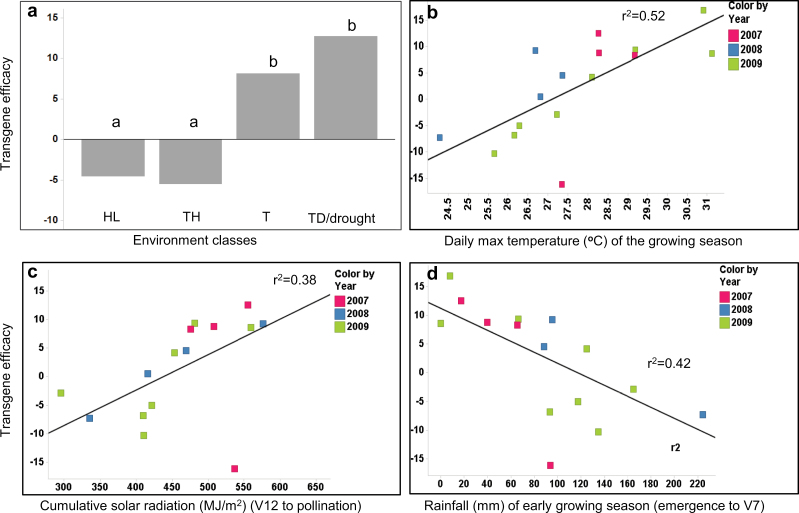
*ZAR1* transgene efficacy in relation to the environmental classes and major environmental factors. (a) Transgene efficacy (Pro_*ZmUBI*_:*ZAR1)* for the four different environmental classes. Transgene efficacy was significantly different under different environmental types, different letters (a, b) indicate statistically significant differences (*P* <0.05 in ANOVA). *ZAR1* transgene showed negative effects on yield under HL and TH environment classes and positive effect on yield under T, TD or drought-stress environments. (b) Transgene efficacy showed a positive relationship with higher daily maximum temperature of the growing season; and (c) with cumulative solar radiation; (d) showed a negative relationship with the amount of rainfall in the early growing season. Transgene efficacy was measured as yield difference of transgenic versus null control in bushel per acre. Yield and environment data were based upon data from three years. HL: High Latitude; TH: Temperate Humid; T: Temperate; TD: Temperate Dry. Note that the environmental classifications refer to the climate at the locations in the years involved; it is not a statement about physical latitude (see Materials and methods).

Further analysis of *ZAR1* transgene-by-environment interaction was conducted using the environment classes and yield data from all three years (without discriminating between alleles). The data showed a positive relationship of *ZAR1* transgene efficacy with warmer and drier environments (T or TD/GFS), and a negative relationship with cooler and wetter environments (TH or HL) (Fig. 4a). Further correlation analysis with major individual environment factors showed that the transgene efficacy positively correlated with higher daily maximum temperature, cumulative solar radiation, and negatively correlated with the amount of rainfall (Fig. 4b, c, d).

### Natural variation of *ZAR1* is associated with drought-tolerance traits and breeding selection

To understand *ZAR1* function in natural variation, its allelic variation in the breeding germplasm of Pioneer was investigated. From a previous genome scan study (Beló *et al.*, 2008), which sequenced 8590 genetic loci across the genome from nearly 600 inbred lines, haplotypes were defined for the maize genome. There are about a dozen haplotypes at the *ZAR1* locus in the founder lines of the germplasm (Braak *et al.*, 2010; van Eeuwijk *et al.*, 2010), however, only two of these are found at high frequency in Pioneer’s modern germplasm. A survey with a subset of 424 Pioneer inbred lines representing elite breeding germplasm showed that these two haplotypes accounted for approximately 95% of the overall allele frequency (see Supplementary Fig. S5 at *JXB* online). The NS lines were predominantly (85%) haplotype 1 (represented by the NS1 allele), and the majority (60%) of SS lines were haplotype 2 (represented by the SS1 allele). A survey of 169 Pioneer’s commercial hybrids identified that the majority (66%) of the hybrids carried the combination of haplotype 1 (NS1 allele) and haplotype 2 (SS1 allele) at the *ZAR1* locus (see Supplementary Fig. S5b at *JXB* online).

**Fig. 5.**
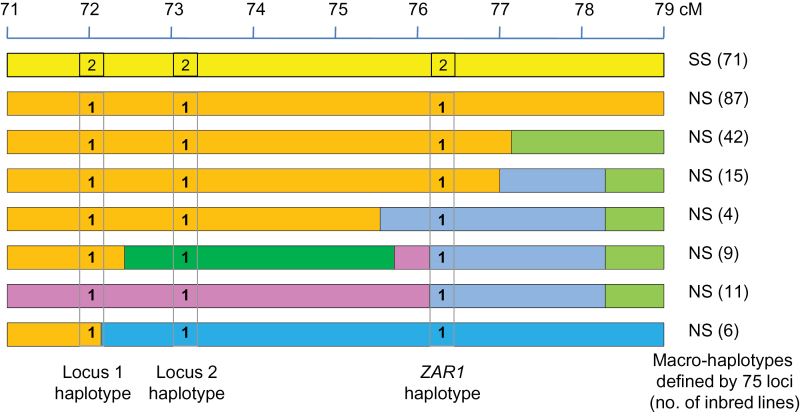
Genetic macro-haplotypes flanking *ZAR1* locus. A macro-haplotype view of *ZAR1* flanking region by using haplotypes of 75 loci from 71 SS and 174 NS lines, including locus 1 and locus 2, with a designated haplotype 2 across SS inbreds and a haplotype 1 across NS inbreds. Colours represent the extension of haplotype similarity across inbreds. Similarity (regions with the same colour) indicates the same founder origin. Breakpoints and mixing of different segments created distinct macro-haplotypes (identified by the combination of segments with different colours). Inbreds that showed no major variation on a segment are therefore the same macro-haplotype (same colour). All SS inbreds showed the same macro-haplotype on that segment. NS inbreds constitute seven distinct macro-haplotypes. Breakpoints within them indicate recombination between the original founder segments during breeding.

To determine if any recombination had occurred nearby the *ZAR1* locus, a subset of 245 inbred lines were chosen, including174 NS and 71 SS, all of which carried the haplotypes 1 and 2 characteristic of the NS and SS heterotic groups, respectively (Fig. 5). Nearly all 75 available loci present in the 8-cM region flanking *ZAR1* were used for macro-haplotype pattern analysis. While all 71 SS inbreds showed no variation on their macro-haplotype pattern, the NS lines had seven macro-haplotypes. The NS macro-haplotypes are formed by juxtaposed founder segments indicating that recombination had taken place in this region (Fig. 5).

Genetic association analysis of the *ZAR1* locus with a number of traits was conducted using populations that were phenotyped for 2007, 2008, and 2009 under drought-stressed conditions. The study involved both heterotic groups, with 234 NS inbred lines, and 133 SS lines, which included all of the inbred lines used for the haplotype analysis. For each heterotic group a single inbred tester was drawn and crossed to all of the lines from the opposite heterotic group to create F_1_ hybrids. The *ZAR1* gene co-localized with QTLs of traits measured under flowering and grain-filling drought conditions. Associations with kernel weight, barrenness, plant height, and staygreen were detected (Table 1). The magnitude of the effect of the *ZAR1* gene was small to moderate and of a similar magnitude for yield-related QTLs detected in elite maize populations (Boer *et al.*, 2007; van Eeuwijk *et al.*, 2010). The two major genetic marker-defined haplotype blocks involving the *ZAR1* locus were identified as favourable, with positive effects on the traits, when the two tester lines used carried the other haplotype, that is, when the SS1 allele and the NS1 allele were in heterozygous combination in the hybrids. Larger numbers of testers would be needed to verify this trend.

**Table 1.** Estimated effect sizes for detected associations of the *ZAR1* gene in the SS and NS heterotic groups for traits in top-cross inbred preference across three years of evaluation under managed drought conditions Effects estimated under flowering stress (FS) and grain-filling stress (GFS) treatments (water deprivation, see Supplementary data, Association experiments, at *JXB* online), are given as having an SS1 and NS1 allele, in heterozygous (NS1/SS1) and homozygous combination in (NS1/NS1) in the NS set; and homozygous (SS1/SS1) and heterozygous combination (SS1/NS1) in the SS set. Numbers in bold and italic indicate effects statistically significant at *P* <0.1. ‘0 .00’ indicated that there is no detected effect of allele substitution.

NS and SS sets and treatments	NS_FS		NS_GFS		SS_FS		SS_GFS	
Testcross genotype	NS1/SS1	NS1/NS1	NS1/SS1	NS1/NS1	SS1/SS1	NS1/SS1	SS1/SS1	NS1/SS1
ASI (GDU)	0.00	0.00	0.00	0.00	0.20	–10.76	0.18	–0.29
Barren plant count (%)	–***0.17***	***–1.90***	1.40	–3.50	0.00	0.00	–0.35	0.09
Kernel no. per ear	1.76	–4.67	1.00	–3.33	–1.64	4.31	0.00	0.00
Kernel wt (grams)	***0.10***	***–-0.01***	0.00	0.00	-0.20	0.16	0.00	0.00
Plant height (inches)	0.20	-0.10	–0.66	0.38	0.00	0.00	***0.77***	***2.12***
Staygreen (1–9 score)	***0.01***	***0.15***	***0.04***	***0.16***	0.00	0.00	0.00	0.00
Grain yield (bushels/acre)	0.00	0.00	-0.60	–7.13	–0.45	0.54	–0.22	2.40

### Functional allelic variation of *ZAR1*

**Fig. 6.**
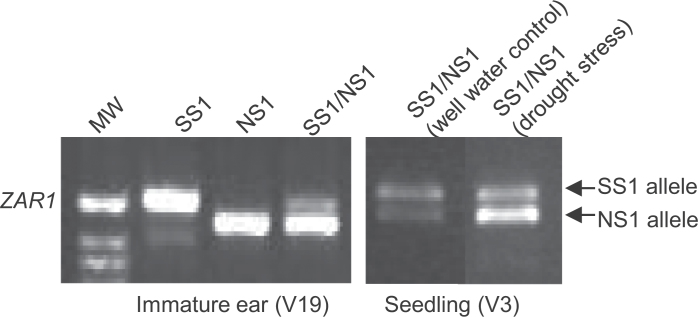
Native allele specific expression of *ZAR1* in hybrid SS1/NS1. Differential allelic expression in the immature ear (V19 stage) and seedling (V3 stage), and response to drought stress in the seedling tissue of the hybrid. Inbred parents SS1 and NS1 are shown as reference control for the SS1 and NS1 alleles. Note the relative allelic expression change from well watered to drought stress. See Supplementary Table S4 for quantitative measurements of allele expression.

To understand possible sources of functional variation of the two major *ZAR1* alleles of SS and NS heterotic groups, the promoter and protein coding regions of the SS1 and NS1 alleles were analysed. Numerous insertions/deletions were found in the promoter region within the 1kb region upstream from the presumed transcription start site (see Supplementary Fig. S6a at *JXB* online), where transcriptional *cis*-regulatory elements usually reside. Allele-specific expression analysis of the SS1/NS1 hybrid showed that SS1 and NS1 alleles were differentially expressed. The allelic expression difference was observed in both immature ear and seedling tissues, and under drought conditions (Fig. 6; see Supplementary Table S4 at *JXB* online). Since both alleles were in a common hybrid, the allelic expression difference is hypothesized to be due to *cis*-regulatory differences (Guo *et al*., 2003, 2004, 2008; Springer and Stupar, 2007). These data provide evidence that the promoter allele variants of *ZAR1* may be functional and account for the differences in temporal, spatial, or stress-responsive expression. Amino acid differences were also found in the protein coding regions between the SS1 and NS alleles (see Supplementary Fig. S6b at *JXB* online). These differences were investigated by protein structural modelling analysis. The alternatives leucine/methionine (L/M) occurred at the most conserved motif, which probably defines secondary protein structure. The variants alanine/glycine (A/G) may allow SS1 to have a highly flexible glycine-glycine-glycine (GGG) loop adjacent to the conserved motif. These amino acid differences could alter the secondary or tertiary structures of the ZAR1 protein, and therefore the protein functions of the SS1 and NS1 alleles.

### Transgene efficacy of *ZAR1* alleles in heterozygous combination

Given that the two major alleles are observed to be favourable in hybrid performance when in the heterozygous combination, vectors were designed to test whether a transgenic heterozygous combination would produce higher transgene efficacy than either allele alone. Since the allelic variation could be due to either expression or protein differences, transformation vectors were built including the native promoter and coding regions of each isolated SS1 and NS1 native allele alone, and a molecular heterozygous allele stack (SS1+NS1).

**Fig. 7.**
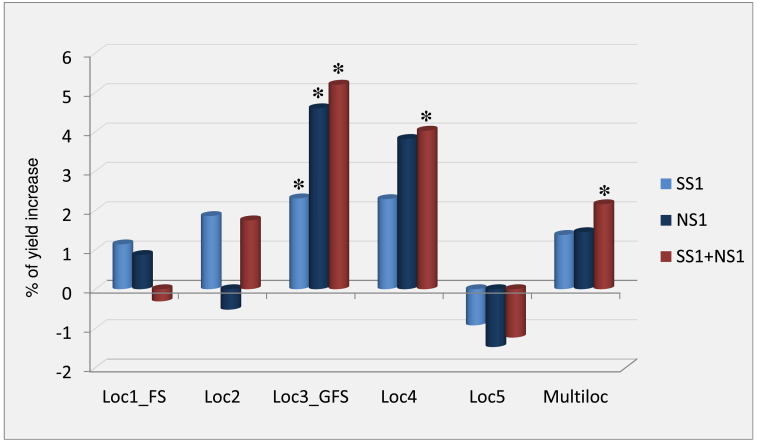
Yield data of *ZAR1* transgenic hybrids with single and stacked heterozygous alleles. Data are the percentage of yield increase of transgenic *ZAR1* hybrids (Pro_*ZmZAR1*_:*ZAR1*) for SS1, NS1, and SS1+NS1 allele variants, respectively, relative to the non-transgenic control in five locations in 2011. Each allele variant contains its own native promoter and protein coding region, with a 35S-enhancer added in the vectors to enhance the level of expression. Multiloc: multi-location analysis of data from all five locations. *x-*axis, testing locations. Loc1 and Loc3 were under Flowering drought Stress (FS), and Grain-Filling drought Stress (GFS), respectively. *y*-axis, percentage of yield increase to control (bulked non transgenic segregants) of each individual construct. For each construct approximately 10 insertion events were tested. * indicates a statistically significant (*P* <0.1). Loc1, Loc2, and Loc3: Yolo, CA; Loc4: York, NE; Loc5: Polk, IA. Locations are by U.S. County, State. Environment classifications were defined by the climate of the season not the physical latitude (see Materials and methods).

Yield testing of these transgenics with multiple insertion events and across five locations in 2011 showed that the individual allele transgenic *ZAR1* plants consistently showed a positive effect. The transgenic plants with the molecularly stacked heterozygous alleles *ZAR1* (SS1+NS1) had a significant yield increase in more locations than either of the single allele transgenics. Multi-location analysis showed that transgenic plants of the heterozygous allele stack had an overall statistically significant yield increase, whereas the transgenic plants of the individual alleles showed a positive impact on yield but were not statistically significant across all environments (Fig. 7; see Supplementary Table S5 at *JXB* online). The data indicate a higher magnitude of yield increase and more stable performance of the transgenic stacked alleles than the single alleles.

This experiment was not intended to distinguish whether the SS1_NS1 positive effect was due to functional allelic complementation of either expression or protein differences. Nonetheless, at least in leaf tissue, total *ZAR1* expression (transgenic plus endogenous allele) was highest in SS1 transgenic plants, whereas NS1 and the SS1+NS1 stack transgenic plants had lower total *ZAR1* expression than SS1 plants, but similar to each other (see Supplementary Fig. S7a–c at *JXB* online). The larger transgene effects (grain yield) did not correlate with higher *ZAR1* leaf tissue expression level (see Supplementary Fig. S7d at *JXB* online). A simple expression dose–response mechanism is therefore not supported by this leaf assay result although it cannot be ruled out. More complex allelic behaviour across spatial, temporal, and environmental response dimensions may exist in the native promoters.

## Discussion

There are few clear and unambiguous instances to date of single transgenes which positively impact complex agronomic traits in crop plants. The significant conclusions of this study are the following: (i) a maize gene *ZAR1*, selected from a class of genes considered ‘intrinsic yield’ genes because of their role in controlling plant organ size, positively impacts transgenic maize size and performance, in part through changes in cell number; (ii) the *ZAR1* single transgene positively impacts complex yield-related trait performance for hybrid maize in field conditions; (iii) the transgene effect shows environmental interactions typical of complex agronomic traits; (iv) functional native allelic variants from a maize breeding programme exhibit different but positive transgene efficacy; and (v) these transgenic alleles positively interact in heterozygous combination, consistent with their participation in hybrid breeding.

### *ZAR1* effects organ size via cell number

Genes involved in plant and organ size control are referred to as intrinsic yield genes (Krizek, 2008; Gonzalez *et al.*, 2009), and they are being studied for their potential to increase biomass and crop yield. It has been demonstrated here that ectopically expressing *ZAR1*, a putative maize orthologue of the *Arabidopsis* ARGOS gene (Hu *et al.*, 2003), increased plant and organ size in maize by multiple metrics. *ZAR1* increased maize plant growth by a faster growth rate and not an extended growth period, as observed for ARGOS in *Arabidopsis*. Consistent with ARGOS gene effects in *Arabidopsis*, the increased plant and organ size was due primarily to increased cell number, not cell size. The maize gene *ZmCNR1*, a negative cell number regulator and orthologue of tomato fruit weight gene *fw2.2* (Frary *et al.*, 2000; Nesbitt and Tanksley, 2001), also affects maize transgenic plant size via cell number (Guo *et al.*, 2010). While *Arabidopsis* ARGOS has been implicated in auxin action (Hu *et al.*, 2003), exactly how it, much less maize *ZAR1,* relates to auxin or other systems remains unknown. On a parallel note, however, two other ARGOS gene family members in *Arabidopsis* have been studied: *ARL* (*ARGOS-like*), which affects plant and organ size by altering cell size, and is involved in the brassinosteroid (BR) pathway (Hu *et al.*, 2006); and the *OSR* (*Organ Size Related*) gene, which affects both cell number and cell size, but is induced by ethylene (Feng *et al.*, 2011).

### *ZAR1* transgene effects on yield and environment interaction

Transgenically over-expressing *ZAR1* in maize not only increased plant and organ growth, but also improved agronomic traits related to drought stress, such as reduced ASI, tip kernel abortion and barrenness. Moreover, the positive effect of the *ZAR1* gene was more pronounced on tip kernel abortion at a higher planting density, and on yield under environments that were hotter and dryer. Crowding or density stresses, and hot and dry environments resulting in drought stress, are known to impact these three agronomic traits similarly. The *ZAR1* yield-related positive effects may, therefore, have environmental interactions that are probably mediated through tolerance to drought and related stresses.

Maize breeders commonly test maize hybrids in multi-environmental trials to evaluate their performance within the target population of environments (TPE) (Löffler *et al.*, 2005; Cooper *et al.*, 2006). Transgenes for insect and weed control traits, with relative mechanistic simplicity, often exhibit effectiveness over broad environmental and geographical regions (Estruch *et al.*, 1997; Dill *et al.*, 2008; Kumar *et al.*, 2008; Duke, 2011). Transgenic effectiveness for more complex agronomic traits with specific environmental conditions remains poorly understood. This study of the *ZAR1* transgene demonstrates environmental conditions interact with the gene’s effect on complex crop performance traits.

### *ZAR1* alleles in transgenics and natural variation

The effects of transgenic *ZAR1* alleles, and the distribution of the allele variants in maize germplasm of Pioneer, presented a parallel between allelic transgene efficacy and allelic effects within breeding germplasm. Breeding selection has changed the genetic make-up of the germplasm (Duvick *et al.*, 2004; Duvick, 2005). Although the underlying mechanisms for the continuous yield gain over the decades of hybrid maize development is essentially unknown, the selection has impacted the allele frequency of many thousands of target genomic regions, and across diverse breeding germplasm (Feng *et al.*, 2006; Hufford *et al.*, 2012; Jiao *et al.*, 2012; van Heerwaarden *et al.*, 2012). Fewer allele types per locus have typically been retained in the elite germplasm pool relative to the original founder pool (Feng *et al.*, 2006). In addition, a subset of allelic variants has become diverged between the SS and NS heterotic groups, from which the bulk of inbred parents of commercial hybrids are derived (Duvick *et al.*, 2004; Duvick, 2005; Feng *et al.*, 2006). The observation that two of the founder *ZAR1* alleles are retained as the predominant alleles in our elite germplasm indicates that the *ZAR1* locus may have been a target of selection in breeding. The selection may be due either to *ZAR1* itself having functionally superior alleles or it being closely linked to other selection targets. Interestingly, a recent study using public breeding germplasm of North American maize has identified a different *ZAR* locus (*ZAR7)* as a target of breeding selection and shown a similar impact on allele frequencies (van Heerwaarden *et al.*, 2012).

The following observations are consistent with functional diversity existing at the *ZAR1* locus having been selected for in hybrid maize heterotic performance. Among 245 elite breeding lines, among both SS and NS germplasm, the *ZAR1* locus seldom or never recombined with its closest markers within the 2 cM flanking range, but instead these other neighbouring markers have recombined across the 245 inbred lines. This indicates that the major *ZAR1* alleles now lie in narrow haplotype blocks surrounded by recombination. Second, yield gains in hybrid maize from breeding are, in considerable part, due to genetic improvement in tolerance to biotic and abiotic stresses (Duvick, 2001; Duvick *et al.*, 2004; Hammer *et al.*, 2009). Our results indicate that *ZAR1* is associated with drought-stress tolerance in both transgenics and in native variation. Third, in hybrid maize development, where heterotic combinability is sought, the selection is based upon yield of top-cross hybrids, not parental inbred lines themselves. Therefore, allelic performance is often evaluated and selected for in hybrids where the alleles are heterozygous. The observation that, at the *ZAR1* locus, the native alleles SS1 and NS1 favour crop performance in the heterozygous combination is consistent with this heterosis. Fourth, both alleles have been retained as the predominant alleles partitioned into Pioneer’s SS and NS heterotic populations representing the female and male lines, respectively. Fifth, when these *ZAR1* alleles were transgenically stacked together in a heterozygous state they outperformed the individual transgenic alleles resembling heterosis. Sixth, the alleles differ in ways that may be functionally significant, in both expression differences and protein structures, providing a possible foundational explanation of their functional differences.

The *ZAR1* alleles’ transgenic effects and the native allelic variation in elite breeding germplasm point to a shared genetic basis governing their genetic contributions as favourable alleles, but also their environmental interaction dependencies. While genetic variation from exotic germplasm or wild species can contribute to the long-term success of conventional breeding efforts, genetic material from the same species (Jacobsen and Schouten, 2007; Orzaez *et al.*, 2010), especially genetic/allelic variations already selected by breeding under specific targeted agronomic environments, such as drought stress or high plant density, may be useful for crop improvement by transgenic and cisgenic approaches.

## Accession numbers

Sequence data from this article can be found in the GenBank databases under the following accession numbers: *ZAR1* (JN252296), *ZAR2* (JN252297), *ZAR3* (JN252298), *ZAR4* (JN252299), *ZAR5* (JN252300), *ZAR6* (JN252304), *ZAR7* (JN252301), *ZAR8* (JN252302), and *ZAR9* (JN252303).

## Supplementary data

Supplementary data can be found at *JXB* online.

Supplementary Table S1. RT-PCR and cloning primers.

Supplementary Table S2. The maize *ZAR* gene family.

Supplementary Table S3. Transgenic hybrid yield of *ZAR1* allele variants relative to control in multiple locations, environment classes and years.

Supplementary Table S4. *ZAR1* allelic expression ratio in the F_1_ hybrid.

Supplementary Table S5. Yield of *ZAR1* transgenic hybrids with single and stacked heterozygous alleles.

Supplementary Fig. S1. The maize *ZAR* gene family.

Supplementary Fig. S2. *ZAR1* transgene effects in inbred plants.

Supplementary Fig. S3. *ZAR1* transgene effects on internode length of inbred plants.

Supplementary Fig. S4. Alteration in cell growth of inbred plants over-expressing *ZAR1*.

Supplementary Fig. 5. Distribution of *ZAR1* alleles in the breeding germplasm and allele combinations in commercial hybrids.

Supplementary Fig. S6. Allelic variation in promoter and protein coding regions of *ZAR1* SS1 and NS1 alleles.

Supplementary Fig. S7. *ZAR1* expression level (both transgene and endogenous gene) in transgenics of different allele configurations.

Supplementary data. Association experiments.

Supplementary Data
