# Supplementary material for: Maize ARGOS1 (ZAR1) transgenic alleles increase hybrid maize yield
Source: J Exp Bot. 2013 Nov 11;65(1):249–60. doi: 10.1093/jxb/ert370 (PMC3883295; doi:10.1093/jxb/ert370)
Supplement: Supplementary Data [file supp_65_1_249__index.html]

Maize ARGOS1 (ZAR1) transgenic alleles increase hybrid maize yield — Maize ARGOS1 (ZAR1) transgenic alleles increase hybrid maize yield — Supplementary Data 

# Maize *ARGOS1* (*ZAR1*) transgenic alleles increase hybrid maize yield

## Supplementary Data

Data files

**Files in this Data Supplement:**

- Supplementary Data - Supplementary Data
- Supplementary Data - Supplementary Data
