# Supplementary material for: Maize ARGOS1 (ZAR1) transgenic alleles increase hybrid maize yield
Source: J Exp Bot. 2013 Nov 11;65(1):249–60. doi: 10.1093/jxb/ert370 (PMC3883295; doi:10.1093/jxb/ert370)
Supplement: Supplementary Data [file supp_ert370_jexbot104562_file001.pdf]

Supplemental Table 1 online. RT-PCR and cloning primers

PCR Primer	Sequence (5' to 3')
ZAR1 RT-PCR_F	TGCATGCGCGGCGCCTTG
ZAR1 RT-PCR_R	CGCCGGTGGACGACGTGGG
ZAR1 Real-time PCR_F	CCGACCTACATGTAGATAATC
ZAR1 Real-time PCR_R	GTCACAAAATCCAAACGAAAG
eIF4g reference real-time PCR_F	TGTCAAATGGCTCGAGGAG
eIF4g reference real-time PCR_R	AACACTTAGGCCAGCATTCG

Supplemental Table 2. The Maize *ZAR* gene family

Member	Protein Length	Chromosome. Bin Location	Maize Loci Public Release v2 5b
ZAR1	146	10.04	GRMZM2G446201
(*ZAR2*)[1]	144	10.04	GRMZM2G446201
ZAR3	105	2.08	GRMZM2G137546
ZAR4	152	6.05	GRMZM2G066029
ZAR5	119	6.	[2]N.A.
ZAR6	64	3.	[3]N.A.
ZAR7	106	5.05	GRMZM2G113583
ZAR8	117	6.05	GRMZM2G354338
ZAR9	126	3.05	GRMZM2G082943

[1]*ZAR1* and *ZAR2* are now believed allelic variants S1 and NS1, respectively, of the gene locus *ZAR1* in the manuscript.
[2]*ZAR5* CDS partially overlaps GRMZM2G175995 near top of Chr. 6.
[3]*ZAR6* CDS region is located in reverse strand 3'UTR of GRMZM2G162250 in B73 Public RefGen v2 position Chr.3: 147591285-147591479.

Supplemental Table 3. Transgenic hybrid yield of *ZAR1* allele variants relative to control in multiple locations, environment classes and year

Year	Location	Environment	(S1) % yield change to control (bushel/acre)
2007	Linn, IA	TH	***-12.14 (133)***
	York, NE	T	***5.76 (140)***
	Tipton, IN	T	***5.86 (149)***
	Bureau, IL	T	***6.1 (161)***

Year	Location	Environment	(S1) % yield change to control (bushel/acre)	(NS1) % yield change to control (bushel/acre)
2008	Linn, IA	TH	0.22 (199)	1.32 (203)
	York, NE	T	-0.15 (215)	0.01 (213)
	Tipton, IN	TH	***-5.39 (169)***	***2.00 (189)***
	Bureau, IL	T	***3.74 (185)***	***5.72 (163)***

Year	Location	Environment	(S1) % yield change to control (bushel/acre)	(NS1) % yield change to control (bushel/acre)
2009	York, NE	HL	4.43 (145)	1.09 (152)
	Saline, MO	HL	***-4.03 (155)***	-2.52 (151)
	Van Buren, IA	HL	***-6.06 (164)***	-2.59 (159)
	Gibson, IN	T	***6.06 (162)***	***4.81 (165)***
	Yolo, CA	GFS	***7.92 (168)***	***15.95 (156)***

Data were analyzed at the construct level. Control is bulked null segregants from all events for each construct. ***Bold and Italic*** values are statistically significant at P<0.1. Positive numbers indicate yield increase, negative numbers yield reduction, compared to control. T: Temperate; TH: Temperate Humid; HL: High Latitude; GFS: Grain Filling drought Stress. Note that the environmental classifications refer to the climate at the locations in the years involved; it is not a statement about physical latitude (see Material and Methods). Locations are by U.S. County, State.

Supplemental Table 4. *ZAR1* allelic expression ratio in the F1 hybrid

Tissue/treatment	Allelic expression ratio in hybrid SS1/NS1	S E
Immature ear	***0.68***	0.068
Seedling (Well watered)	1.03	0.179
Seedling (Drought stress)	***0.79***	0.060

Data are based upon average of three biological replicates, and three plants per replicate. Bold and italic type indicate allelic ratio statistically significant from 1.0 (P<0.05) in t-test.

Supplemental Table 5. Yield of *ZAR1* transgenic hybrids with single and stacked heterozygous alleles

Year	Location	(S1) % yield change to control (bushel/acre)	(NS1) % yield change to control (bushel/acre)	(S1+NS1) % yield change to control (bushel/acre)
2011	Yolo, CA (FS)	1.14 (145)	0.87 (148)	-0.31 (145)
	Yolo, CA	1.86 (163)	-0.52 (160)	1.75 (163)
	Yolo, CA (GFS)	***2.31 (201)***	***4.60 (205)***	***5.20 (201)***
	York, NE	2.29 (157)	3.82 (146)	***4.03 (157)***
	Polk, IA	-0.92 (160)	-1.47 (173)	-1.23 (160)
	Multi-location	1.38 (165)	1.45 (167)	***2.16 (165)***

Data were analyzed at the construct level. Control is bulked null segregants from all events for each construct. ***Bold and Italic*** are statistically significant at $P<0.1$. Positive numbers indicate yield increase, negative numbers yield reduction, compared to control. FS: Flowering drought Stress; GFS: Grain Filling drought Stress. Locations are by U.S. County, State.

a

```
                1                                                                                                                                                                162
ZAR1    (1) ---MSTTRPEDTQQLINSAAASPNRSAPSAAPSDMERGSGTAASSSRASTTSHSHQRATHRVVEEEEEEEEPSSSRGGGSLCSGYLSLP-ALLLVGVTASLVILPLVLPPLPPPPSMLMLVPVAMLLLLLVLAFMPTSSTGGRGGTGPTYM-----------
ZAR2    (1) ----MSAGPEDTQQLINSAAASPNRSAPSAAPSDMERGSGTAASSSRASTTSHSHQRATHRVVEEEEEEE-PSSSRGAGSLCSGYLSLP-ALLLVGVTASLVILPLVLPPLPPPPSLLMLVPVAMLLLLLVLAFMPTSSTGGRGGTGPTYM-----------
ZAR3    (1) --------------------------------------MASRSSAMEGGAAIQRRNAVKRHLQQRQQEADFLDKK---VIASTYFSIGAFLVLACLTVSLLILPLVLPPLPPPPSLLLWLPVCLLVLLVVLA-FMPTDVRSMASSYL---------------
ZAR4    (1) MCRGLPTPAPAPALQFQSQDCSRQQRGTTQAPPGRASESVRACMAAERKAASRPAACGRMRGAEGAKPRGRQAKAARAPPGQGYFTAGLAALFLCLTTLLVFLPLVLPPLPPPPLLLLLVPVGLMAVLLALALVPSDGRAAAAAVASSSCVC----------
ZAR5    (1) -------------------------------MHLLDDLRQDRGGAAAHTGSRSRKPPPPLAAAAAAAAGVPAGSS--TAATATHLGPEAAALLACVTATLLLLPLVLPPLPPPPPLLLLVPVAIFAVLLLLV-LLPSDARAAVATPTSSASYL---------
ZAR6    (1) -----------------------------------------------------------------------------------MSKRVLMMLLAATVILLCLPLVLPPLPPPPLFLLFVPVVMMLLLFSLV-FFPSNHCPCSSPTFTQ------------
ZAR7    (1) ------------------------------------------MPSSSQTPPPPVGRTAAHGGRHKHDDDDPSTPRG---FCAKYFSRESCLLLALVTVLLVVLPLVLPPLPAPPLALLLVPVAMLAVLLVLALMPAAAGGRNEAVDPASYL----------
ZAR8    (1) -------------------------------------------MMLHCTFAISEAPARALALGQVSVMRAMPQEEEAAVATTTMAGGKVAALLATAAALLLLLPLALPPLPPPPTQLLFVPVVLLLLVASLA-FCPAATSSPSPMHAADHGSFGTTGSPHLC
ZAR9    (1) ------------------MPVASSLMAMELETDQLAWAEQQRQQNRRQTMVVCRKSDAAVAKGQQRQNASPPSPK---PPPAGGLSAEAFLVLACVAVSLIVLPLVLPPLSPPPPLLLLVPVCLLLLLAALATFVPSDVRSMPSSNL---------------
```

b

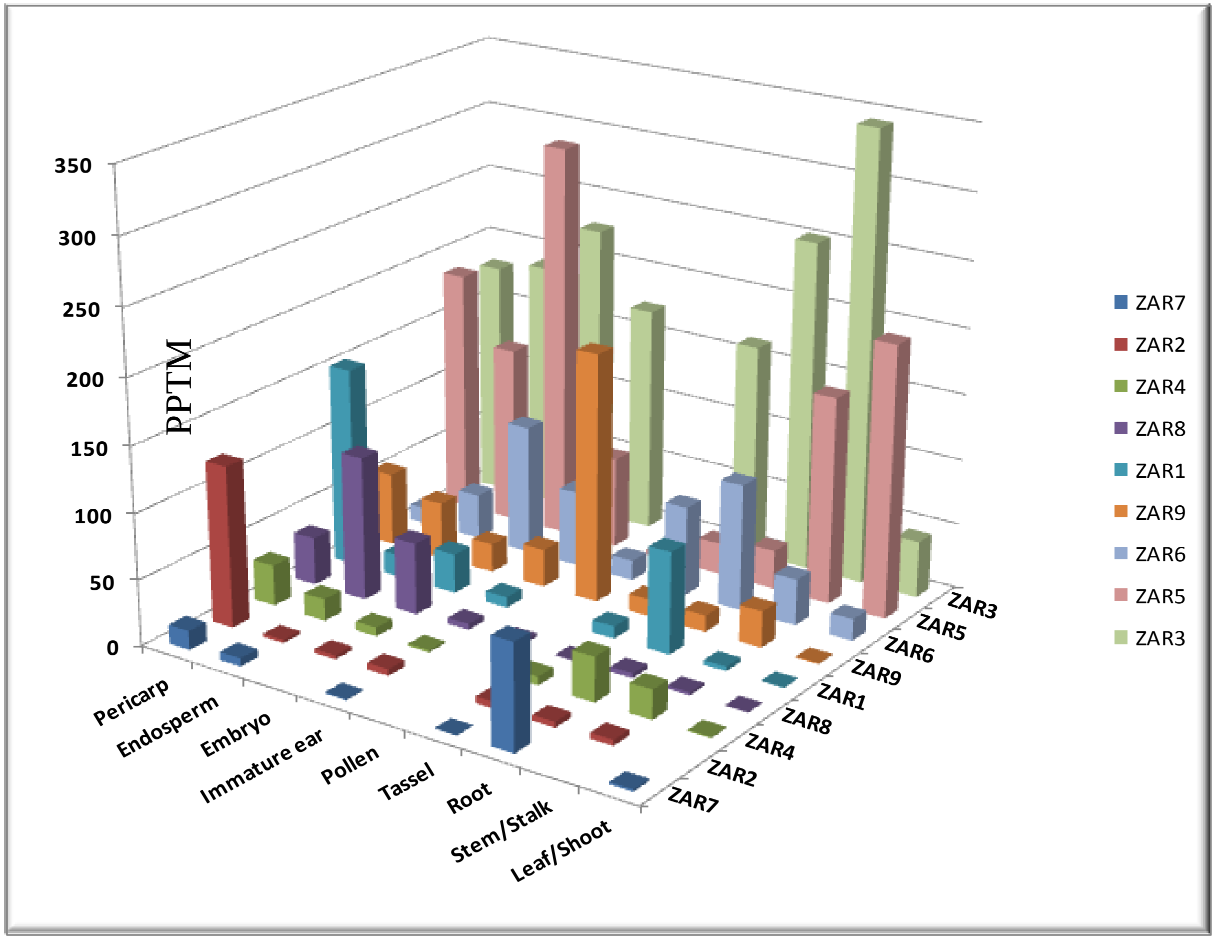

Supplemental Figure 1. The maize *ZAR* gene family

a. Protein sequence alignment of the *ZAR* family members. Bold and shading indicate consensus among the family members. **b.** Tissue expression patterns of the *ZAR* gene family in maize based upon our Solexa/RNA profile databases. The transcript level for each gene is shown as a mean frequency in parts per ten million (pptm).

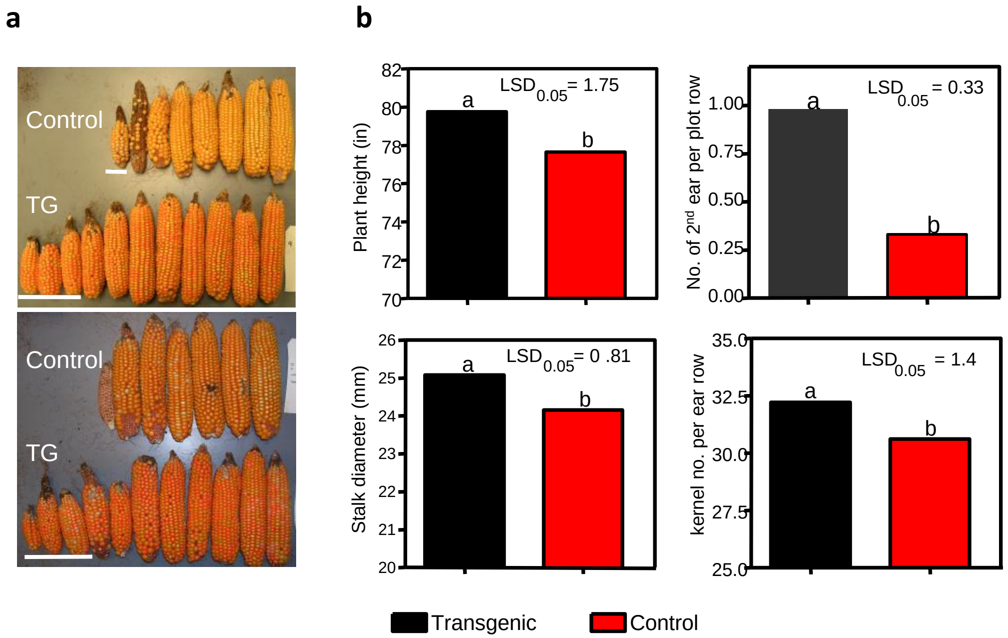

Supplemental Figure 2. *ZAR1* transgene effects in inbred plants

a. Primary and secondary ear growth of inbred plants overexpressing *ZAR1* (Pro$_{ZmUBI}$:*ZAR1)*. Shown are primary and secondary ears (underlined) harvested from all plants of the entire rows (targeted 14 plants per row). **b.** Vegetative growth and reproductive growth measurements of transgenic plants. Data are from average of 9 events, 5 plants per replicate, 3 replicates per event. TG: transgenic; control: null segregant of the transgenic plants, Different letters “a” vs. “b” indicates a statistically significant difference at the given LSD.

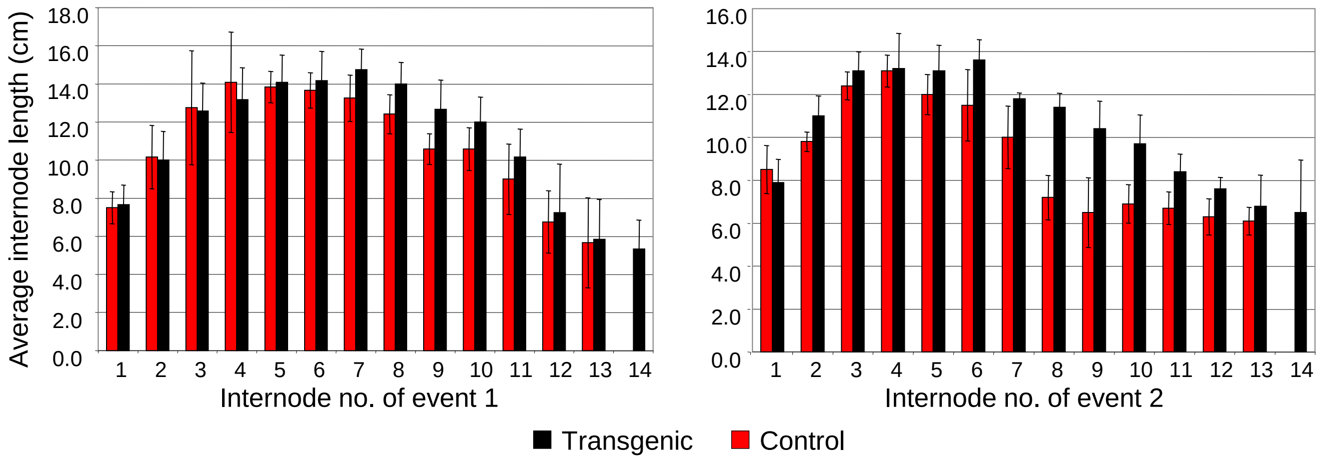

Supplemental Figure 3. *ZAR1* transgene effects on internode length of inbred plants

Two individual insertion events were grown in the greenhouse. The plants were characterized for the number and length of the internodes. Internode length was measured by the distance between nodes, with the brace roots considered the first node, and the base of the tassel the final node (no. 13 or 14). Data represent the average of 5 plants in each event. X-axis, internode number from base, y-axis, length of the internode. Data was collected from mature plants grown in the greenhouse. Bars in the graph show the standard error.

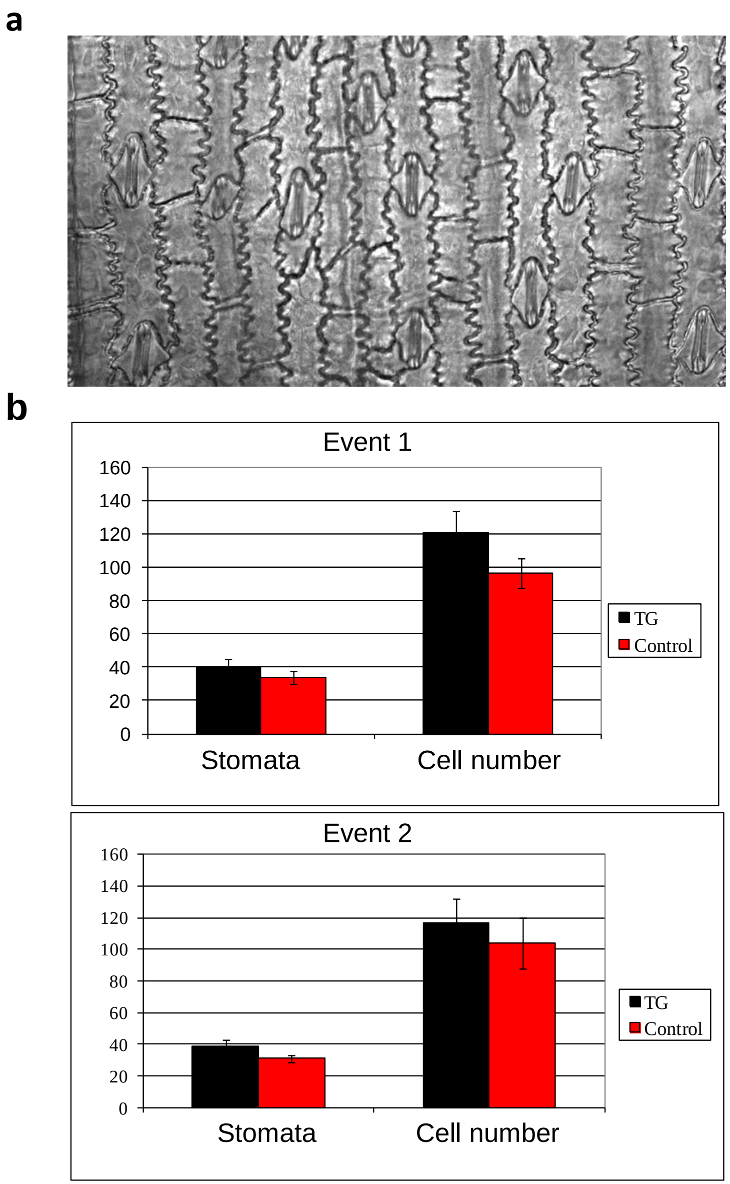

Supplemental Figure 4. Alteration in cell growth of inbred plants overexpressing *ZAR1*

a. Example image of the leaf epidermis from a control plant. **b**. Leaf epidermal cell number and stomata counts per unit leaf area ($1mm^2$), from two individual transgenic events.(Pro_{ZmUBI}:*ZAR1)*. Data were collected from 5 transgenic and non transgenic control plants, respectively, per event. Bars in the graph show the standard error. Y axis, number counts per unit area ($1mm^2$).

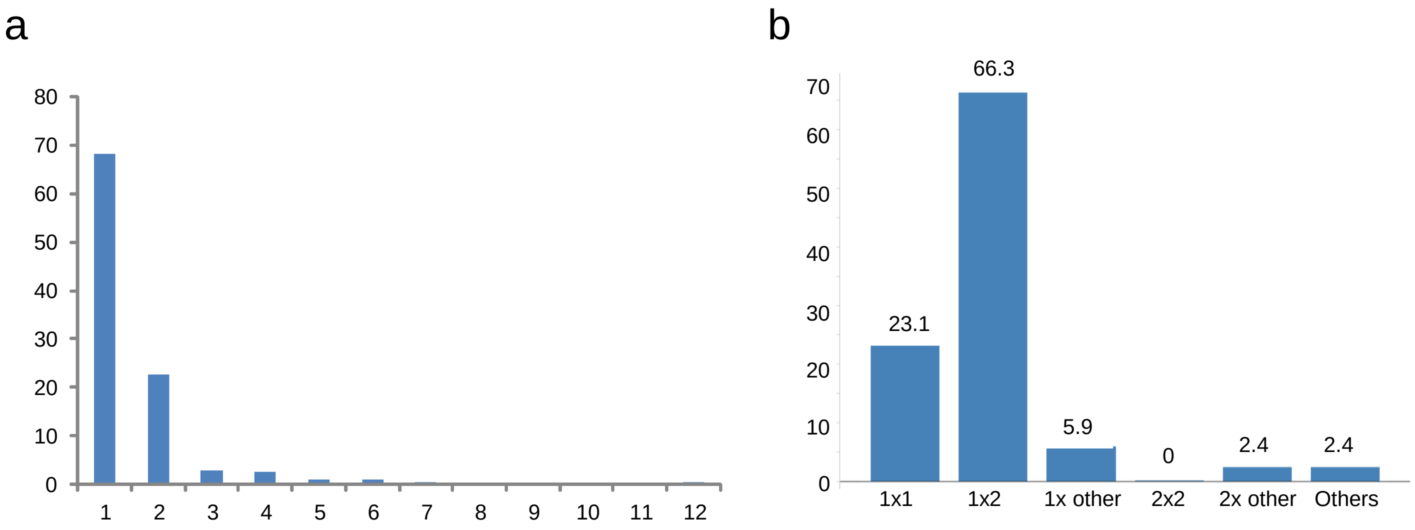

Supplemental Figure 5. Distribution of *ZAR1* alleles in the breeding germplasm and allele combinations in commercial hybrids

a. *ZAR1* allele (represented by haplotypes) frequency in the elite breeding germplasm. Haplotype 1 and 2 represent approximately 95% of the total allele frequency. b. Percentage of different allele (haplotype) combinations at the *ZAR1* locus in a diverse sample of Pioneer commercial hybrids (released during the period 1987-2011) where parental data for *ZAR1* locus was available. X axis, *ZAR1* haplotypes in the germplasm (a) or haplotype combinations in the hybrids (b). Y axis, percentage of total alleles (a) or allele combinations (b). 1 represents haplotype 1 (NS1 allele); 2 represents haplotype 2 (SS1 allele); Others represent haplotypes other than 1 and 2. Y-axis, percent of the total number of inbreds or hybrids surveyed, respectively.

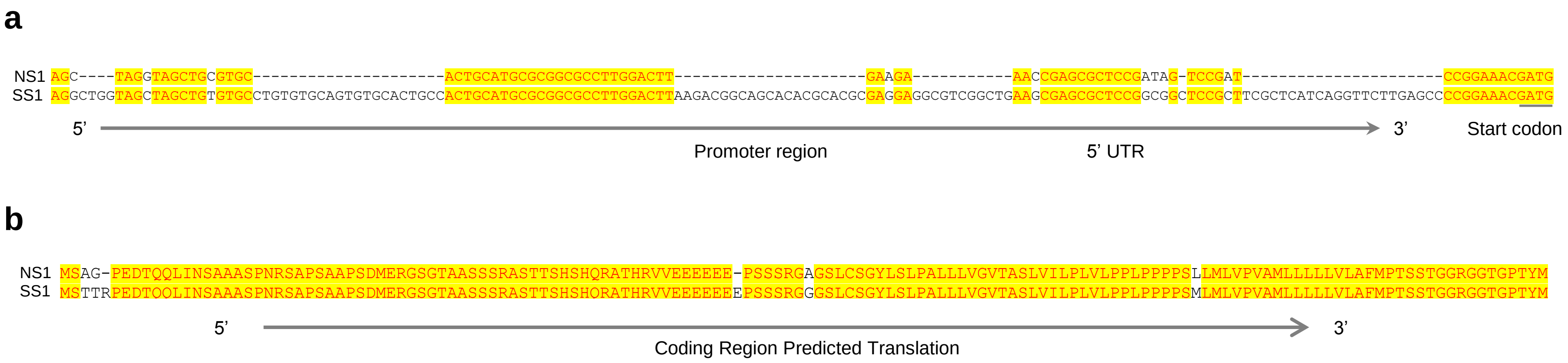

C

SS1 allele promoter (1376 bp)

tgattaagctagtacatcgcccctagatttccggcagaattaagaaaaccgcgggcagcagagccgatgccgatggcaacaaagaagaaggggctgttggtactgcagccgcagtctataaagataaaaattgtagaagtagtaggaagcttagccggagctggcatggcaggctgctgctgagtgagcagcgg
tgggggcctcctggctggcgcgtggaaaaacccgagcaaatggcagcgtgaagcacgtccgagactgaggtcaggcgtcgggcgggggttgcggccaggggagacgaatgaacccctgcccccgcctggatcccatcgcaaaagccccctccccctctccgccttcgcgcatattatattcgcgcaccatcg
cagcaacttgcacgggcgccgatgactagttgcgccagatgcactgcatctgctcggcgtggtgcctccaacgtccaacccctcttcctcttgtctctcgtctacctctcttctgcccctctgcgtccgtgtctccatcgtcgtcgctgcgtgaggttgacgacgaccagtcacaggatctgttcgttcctcatgcgaccca
gctagctaaaactggcatgcatggacatgctacgctgctgcgtcaatccatctcaccaggtacgctgctgtacatgctgctacgagcctacgatcgatagcagtctgtgccttcctttgctcgatgccgatgtttatctgcatgtgatcgtattcgtatgcacggccctccgccctctcaagctgagtgctttttggtgggcc
catcgtcctatatacgctcatcagttcactgacgacgatataacgactgttggggttcagaaactacatattgtggtgctcgcccgatctctttcttgtatattcttcttattattagtctctctctctctgaaagaacaaggaactagatgtcttgttttgtgcctcctactataccttttgcgtgtttttcttgcttttgtccatggcttttca
ccggtctgctgggtgaagtaatttacacgcatgtcttacgcacgcgctccttcagttgtccgcatatctgatcataacatcgcttcattcatgtgctgacgagatatttttcgccgccgagactgcagtgctagctagctagatctggcctgattcgccgatcgagcggtggtgagacggagtgcttcagctcaaagactgc
tagtggtaggctggtagctagctgtgtgcctgtgtgcagtgtgcactgccactgcatgcgcggcgccttggacttaagacggcagcacacgcacgcgaggaggcgtcggctgaagcgagcgctccggcggctccgcttcgctcatcaggttcttgagccccggaaacg

NS1 allele promoter (1724 bp)

ataaattgattaagctagtacatcgcccctagatttccggcagaattaagaaaaccgcgggcagcagagccgatgccgatggcaacaaagaagaattggtactgcagccgcagtctataaagataaaaattgtagaagtagtaggaaggttaggctatccgcagcggttccctctaaatttttccccctatatcacttttct
gggtcacatcattaatagtttatcccctactttttcatccccgcagcggttccccccttaatactcccccctataccccactacaatataaaatattattttctatacctactttttatctactatcaatttttcttctactattaattcaaaacgggcccacacgaacagtgcaagggggagctcgcgcgcgcgctgcagctggcggga
gagagaggagtcccgccgtgtagagcgcgtgctagaggcagccgctgcagcgctagggagtgccctgtagccggcatgcaagctacagggcaaggggaggggggaagcgcgcggtgtcccttgcggacagtcttagccggagctggcatggcaggctgctgagtgagcagcggtgggggcctcctggct
ggcgcgtggaaaaacccgagcaaatggcagcgtgaagcacgtccgagactgaggtcaggcgtcgggcggggggggcggccaggggagacgaatgaacccctcccccccgcctggatcccatcgcaaaagccccccttcccctccccccctctccgccttggcgcatattatattcgcgcaccatcgccgcaacttg
cacgggcgccgatgactagttgcgccagatgcactgcatctgctcggcgtggtgcctccaacgtccaacccctcttgtctctcgtctacctctcttctgcccctctgcgtccgtgtctccctcgtcgtcgctgcgtgaggttgacgacgaccagtcacaggatctgttcgttcctcatgcgacccagctagctaaaactgg
catgcatggacatgctacgctgctgcgtcaatccatctcaccagcaggtacgctgctgtacatgctgctacgagcctacgatcgatagcagtctatgcctttgctcgatgccgatgtttatctgcatgtgatcgtattcgtatgcacggccctctcaagctgagtgctttttggtgggtccatcgtcctatatatacgctcatca
gttcactgacgacgatataacgattgttggggttcagaaactacatatggtggtgctcgcccgatctctttcttgtatattcttcttattattagtctacttctctctctctctctctttctgaaagaacaaggaactagatgtcttgtcttgtgcctcctactatacctttacgtgtttttcttccttttgtcgtccatggcttttcaccggt
ctgctgggtgaagtgaagtaatttacacgcatgtcttacgcacgcgctccttcagttgtccgcatatctgatcataacatcgcttcattcatgtgctgacgagatatttttcgccgccgagactgcagtgctagctagctagatctggcctgattcgccgatcgagcggtcgccggtcagagactcagagttcatgagacg
gagtgcttcagctcaaagactgctagtggtagctaggtagctgcgtgcactgcatgcgcggcgccttggacttgaagaaaccgagcgctccgatagtccgatccggaaacg

Supplemental Figure 6. Allelic variation in promoter and protein coding regions of *ZAR1* SS1 and NS1 alleles

a. Sequence alignment showing the sequence differences and insertions/deletions in the proximate promoter region between the two alleles. **b**. Amino acid sequence changes or insertions/deletions in the entire coding region between the two alleles. **c.** Promoter sequences for SS1 and NS1 alleles.

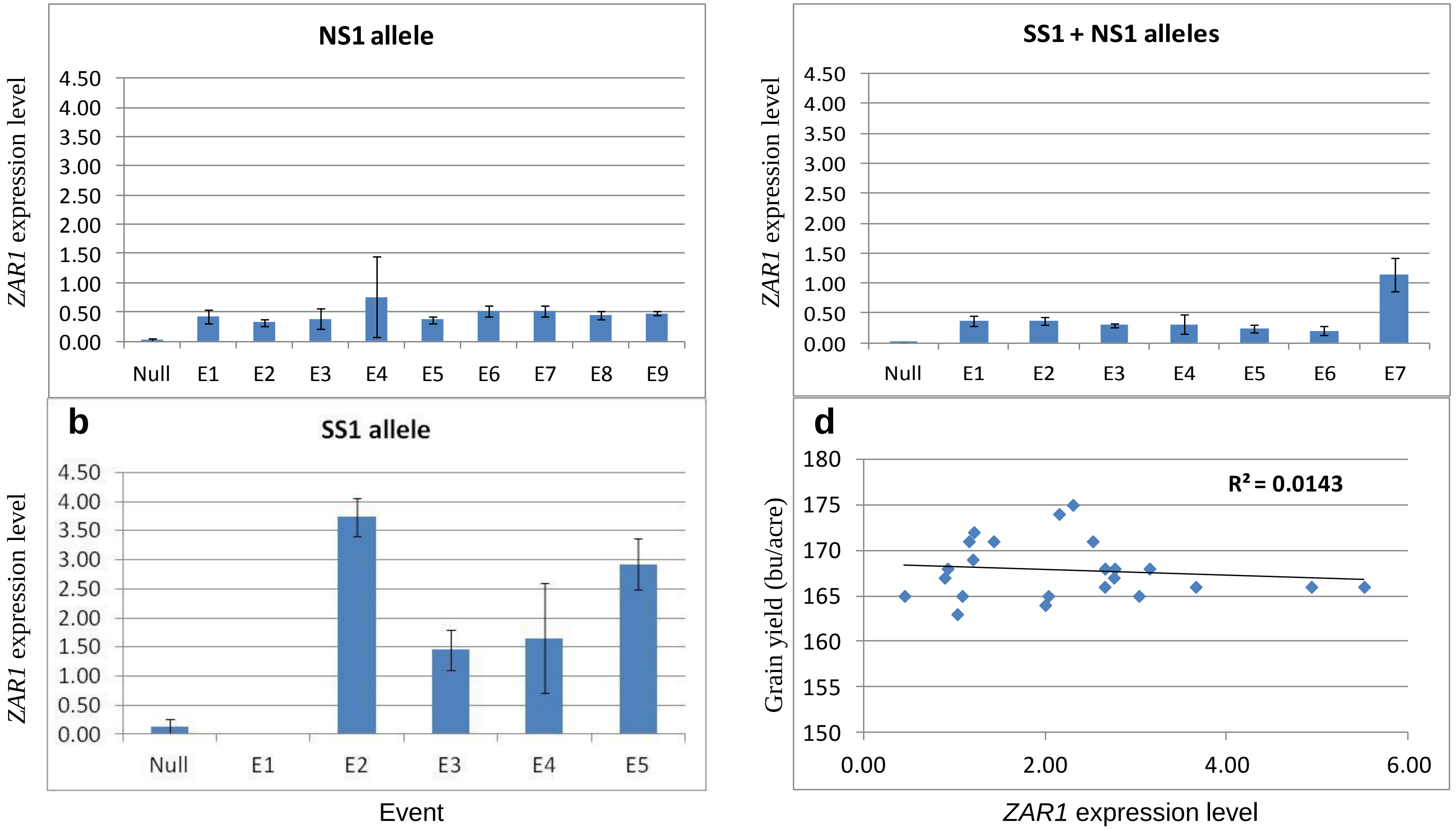

Supplemental Figure 7. *ZAR1* expression level (both transgene and endogenous gene) in transgenics of different allele configurations.

All three vectors contain the *ZAR1* gene with its corresponding native promoter allele variants and a 35S enhancer upstream to enhance the expression level and minimize altering the native promoters' specificity in gene expression regulation (see Material and Methods). Transgenic plants of stacked alleles (SS1+NS1) did not show higher level of total *ZAR1* expression (even with extra allele) as compared to transgenics of SS1 or NS1 allele (**a, b, c**). The transgenic plants of the S1 allele instead, showed higher *ZAR1* expression level. In each individual transgenic event, average expression and standard error bars were based upon 3 biological replicates, each consisting of 3 plants. Null is the non transgenic segregant for each construct. X-axis, individual events, Y-axis, *ZAR1* transcript level in arbitrary units from real time qRT-PCR. **d**. Correlation of grain yield with *ZAR1* expression level. Same data from above was used. X-axis, the *ZAR1* transcript level in arbitrary units from real time qRT-PCR analysis. Y-axis, grain yield in bushel per acre.
