# Supplementary material for: Maize ARGOS1 (ZAR1) transgenic alleles increase hybrid maize yield
Source: J Exp Bot. 2013 Nov 11;65(1):249–60. doi: 10.1093/jxb/ert370 (PMC3883295; doi:10.1093/jxb/ert370)
Supplement: Supplementary Data [file supp_ert370_jexbot104562_file002.pdf]

**Association experiments:**

To conduct a drought association analysis, sets of SS and NS inbreds were sampled from the Pioneer germplasm collection to represent active elite inbreds and key ancestral inbreds tracing back to the founders of the current SS and NS heterotic groups. Hybrid seed was created for each of the inbreds by crossing the inbreds to an appropriate tester from the complementary heterotic group. The hybrid seed was used for all trait measurements. The SS and NS sets of hybrids were evaluated in experiments for grain yield and agronomic trait phenotypes under drought conditions, and over a period of three years from 2003 to 2005 at the Pioneer Viluco Chile station. There was no in season rainfall during the conduct of the experiments. Therefore, all water inputs were managed through irrigation. Each of the SS and NS sets of hybrids was evaluated in a combination of water management treatments following the methodology described in Campos et al. (2006); two stress treatments referred to as flowering stress and grain-filling stress, and a well-watered control treatment. For the well-watered control treatment (WW) water was supplied to maintain high soil water content throughout the season. For the flowering stress treatment (FS) water was withheld to impose a water deficit during the flowering period. For the grain-filling stress treatment (GFS) water was withheld after flowering to impose a water deficit during the grain-filling period. The FS treatment was implemented in all three years, the GFS treatment was implemented in 2003 and 2005, and the WW treatment was implemented in 2003 and 2004. The FS and GFS treatments were designed as row-column designs with two replicates. The WW treatment was conducted as a 2-replicate row-column design in 2003 and as a single replicate augmented design in 2004. All data were collected from 2-row experimental plots. The row column coordinates of all experiments were used to conduct a spatial analysis in combination with the experimental design (Gilmour et al., 1997). A combined analysis across years and treatments was conducted using ASREML (Gilmour et al., 2009, www.vsni.co.uk) and Best Linear Unbiased Predictors (BLUPs) were computed for all traits in all treatment conditions. The BLUPs for the traits for each inbred, based on the hybrid performance, from the FS and GFS treatments from the combined analysis, were used as the trait phenotypes for the association analysis.

The model for evaluating the effect of *ZAR1* conditional on background marker effects was thus be represented as

Y ~ mu + A1 + b1 + b2 + … + bN (1)

Where Y is the trait phenotype (i.e., BLUP value), A1 is the marker genotype at the *ZAR1* locus, and b1...bN are the marker genotypes at the N background co-factor markers. Significance testing was conducted by comparison of Equation 1 with a base model that excluded the effect of *ZAR1*:

Y ~ mu + b1 + b2 + … + bN (2)

The *ZAR1* locus was determined to have a significant effect on trait phenotypes if the difference in log-likelihood between models 1 and 2 was larger than a chi-square statistic with 2 degrees of freedom. Trait effects for the *ZAR1* locus were estimated based upon Equation 1.

For the combined analysis, environment effects were modeled as fixed effects and genotype and genotype-by-environment effects were random effects. A genotype-by-environment analysis was performed to estimate genetic variances for each environment and genetic correlations between environments.

**References:**

**Campos H, Cooper M, Edmeades GO, Löffler C, Schussler JR, Ibañez M.** 2006. Changes in drought tolerance in maize associated with fifty years of breeding for yield in the U.S. Corn Belt. *Maydica* 51, 369-381.

**Gilmour AR, Cullis BR, Verbyla AP.** 1997. Accounting for natural and extraneous variation in the analysis of field experiments. *Journal of Agricultural, Biological, and Environmental Statistics* **2**: 269-293.

**Gilmour AR, Gogel BJ, Cullis BR, Thompson R.** 2009. ASReml user guide release 3.0 VSN International Ltd, Hemel Hempstead, HP1 1ES, UK.
